# An *in vitro* evaluation of drugs used in the Kenyan ART program

**DOI:** 10.11604/pamj.2016.23.134.7157

**Published:** 2016-03-25

**Authors:** Joseph Muriuki, Zipporah Ng'ang'a, Raphael Lihana, Raphael Lwembe, Joseph Mwangi, Matilu Mwau

**Affiliations:** 1 Centre for Virus Research (CVR), Kenya Medical research Institute (KEMRI), Nairobi Kenya; 2 Institute of Tropical Medicine and Infectious Diseases (ITROMID), Jomo Kenyatta University of Agriculture and Technology (JKUAT) Juja Kenya; 3 Centre for Parasitic and Infectious Diseases (CIPD), Kenya Medical research Institute (KEMRI), Busia Kenya

**Keywords:** HIV, susceptibility, anti retroviral therapy

## Abstract

The majority of anti-HIV drug susceptibility tests have been performed on subtype B HIV-1 strains, since these are the most prevalent in countries designing, testing, and manufacturing the current anti-HIV agents. The increasing global spread of HIV subtype highlights the need to determine the activity of anti-HIV drugs against subtypes of HIV other than subtype B. Furthermore an increasing number of individuals infected with many of the non subtype B virus strains now receive antiretroviral therapy because of rollout programs in developing countries as well as increasing migration to the developed world. The phenotypic susceptibility of two laboratory strains HIV-1JFRL and HIV-1IIIB (representing subtype B) and two clinical isolates HIV-1_04RTA_ and HIV-1_025RTA_ (representing subtypes A and D respectively) was determined. The *in vitro* drug susceptibility testing of the isolates was carried out in C8166 cell line and in peripheral blood mononuclear cells (PBMC_s_). The study revealed that the drugs used in the Kenyan national ART program inhibited HIV-1 replication in-vitro as their inhibitory concentrations (IC_50_) compared well with the standard Inhibitory concentration values. The results also suggest a biochemical similarity of the reverse transcriptase (RT) and protease enzymes from these subtypes despite the divergence at the genetic level. The findings suggest that similar clinical benefits of antiviral therapy obtain in persons infected with other subtypes of HIV-1other than subtype B and that the generic drugs used in the national ART program in Kenya are as efficacious as branded drugs in inhibiting HIV replication in vitro despite the limited number of the viruses studied.

## Introduction

Antiretroviral drug design, resistance research, and interpretation systems have been largely based on HIV-1 subtype B viruses, which have historically been the most prevalent subtype in North America, Western Europe and Australia even though subtype B viruses account for only about 12% of the worldwide HIV-1 infections. Subtype C viruses are the most prevalent, accounting for about 50% of cases [1]. Despite this diversity and prevalence, non-subtype B (NSB) viruses remain largely uncharacterized [2]. An increasing number of individuals who are infected with many of the non– subtype B virus strains now receive antiretroviral therapy because of rollout programs in the resource-limited world and because of increasing migration to the developed world, particularly to countries in Europe. In Kenya approximately 500,000 adults and 40,000 children were on ART by end of 2011[3].

Currently, there are 6 classes of ARV agents with 25 drugs approved for single drug treatment and 12 as fixed dose combinations (FDCs) by the United States food and drugs administration US-FDA [4]. These drugs target 4 distinct proteins namely; host cell receptor, reverse transcriptase, integrase and protease to retard HIV replication. The conventional classes of ARVs are nucleoside reverse transcriptase inhibitors (NRTIs), non-nucleoside reverse transcriptase inhibitors (NNRTIs) and protease inhibitor (PIs). The target of NNRTIs is the same as NRTIs (viral reverse transcriptase). They inhibit HIV-1 RT by binding and inducing the formation of a hydrophobic pocket proximal to, but not overlapping the active site [5] Protease inhibitors (PIs) the third class of approved ARVs function by blocking proteolysis of the viral polyprotein, a step required for the production of infectious viral particles [6]. In the presence of a PI, only defective viral particles, unable to infect new cells, are produced. PIs are used in combination with two nRTIs as part of initial ART. Because they have limited bioavailability, they are co-administered with a pharmacologic “booster”, a low and virologically inactive dose of ritonavir a pharmacokinetic enhancer [7].

## Methods

### Test compounds and viruses

Five drugs used in the national ART programme namely AZT, ABC, EFV, LPV/r, and NVP/3TC/d4T were obtained from the *Liverpool VCT Care and Treatment*, a comprehensive care and treatment Centre in Nairobi in tablets form. Clinical isolates HIV-1 _04RTA_ and HIV-1_025RTA_ *(Genebank ascension numbers* JF829686/A1 and JF829689/D respectively) were sourced from the Centre for virus research HIV laboratory strains bank.

### Drug susceptibility assays

#### For CXCR4 Viruses (HIV-1_IIIB_, HIV-1_04RTA_ and HIV-1_025 RTA_)

Antiviral assays were performed as previously described [8] with some modification. Briefly, C8166 cells growing in the logarithmic phase were inoculated with viral stock (HIV-1_IIIB_, HIV-1_04RTA_ and HIV-1 _025RTA_) at a MOI of 0.001 and incubated at 37°C in a CO_2_ incubator for 1h followed by washing in PBS. The cells were plated (1 ×10^5^/ml) in the absence or presence of ARVs prepared in 5 fold serial dilutions in culture medium and DMSO in a final volume of 200µl. Control wells containing cells and virus were co incubated on each plate. After a 3-5day incubation the wells were checked for signs of syncytium each day and once detected in control wells with no drugs, the supernatants were harvested and tested for viral growth using an ELISA specific for the P24 antigen of HIV-1 (*Biomereaux, France*) as described by the manufacturer. The percentage inhibition of viral growth compared with control wells without drugs was calculated. The IC_50_ was determined from the dose response curves generated. All experiments were performed at least twice and mean percentage inhibition determined by the formulae;

% Suppression = (Control P24 (pg/ml) – Test P24 (pg/ml) x 100)/Control P24 (pg/ml)

#### Anti-HIV-1 assay for JRFL strain (R5)

The antiviral assay was performed as previously described by Japour et al [9] with some modifications. PBMCs from healthy donors were obtained by density gradient centrifugation using ficol paque (*Amersham Biosciences, Sweden*). PBMCs obtained were washed 3 times in PBS and cell count done using the trypan blue dye exclusion test. The PBMCs were pelleted in a 50 ml falcon tube and held in the incubator for 3 hours for virus adsorption to take place; they were then washed in PBS. The cell pellet was re suspended in fresh complete RPMI 1640 medium supplemented with 50 *U/ml human recombinant* IL-2 (Gibco, USA) and seeded into the 96 well microtitre plate at a concentration of 10^5^ cells /ml. Medium was added to duplicate wells up to a volume of 200µl/well and plate incubated. On day 4 half the medium was removed from the wells and replaced with same volume of fresh stimulated PBMCs containing the same concentration of the drugs as originally used. On day 7, supernatants from wells were harvested and production of HIV P24 both in the well with the test compounds as well as the control culture quantitatively determined by reading the absorbance at 490nm/630nm in the ELISA reader following the manufacturers’ instructions. HIV suppression was calculated using the formulae shown above.

#### MTT cytotoxicity assay

The cellular toxicity of compounds on C8166 cells was assessed by MTT colorimetric assay as described previously [10]. Briefly, 100ul of C8166 cells (1x 10^5^/ml) were seeded on a 96 well microtiter plate and 100ul of various concentrations of the test compounds were added. The plate was incubated at 37°C in a humidified CO2 incubator for 72 h. 100µl of the cell supernatant was discarded from each well and 20ul of MTT reagent (*Roche, Indianapolis USA*) was added and incubated for 4h, followed by 100µl of solubilization buffer to dissolve the formazan crystals. After the crystals were dissolved completely, the plates were read on an ELISA reader (*Lab systems, USA*) at 540nm. The percentage cell viability (CV) was calculated manually from absorbance values using the formula:

CV = (Average absorbance of duplicate drug wells x 100)/Average absorbance of control wells

A dose-response curve was plotted to enable the calculation of the concentrations that reduced the number of viable cells by 50% (CC_50_).

## Results

**Figure 1:**
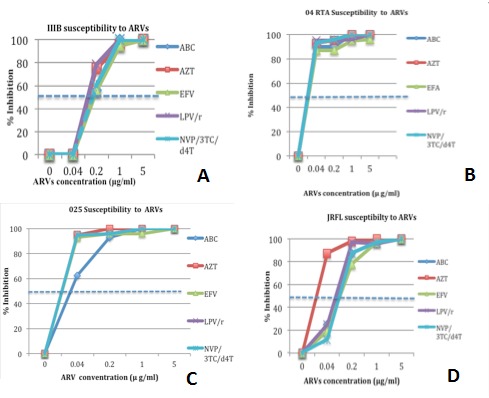
Drug susceptibility curves for AZT, ABC and EFV. LPV/r and NVP/3TC/d4T against (A) HIVI_IIB_, (B) HIV 04RTA, © HIV_025RTA_ and HIV-1_JRFL_ (D). Each dilution of the drug was tested in duplicate in C8166 cell line for the three X4 viruses and in PBMCs for the JRFL (R5). Results represent the means of two independent experiments; the figure shows percent inhibition of P24 antigen production in culture supernatants. The intersection of horizontal broken lines denotes the 50% inhibitory concentration [IC 50] of each drug

**Figure 2:**
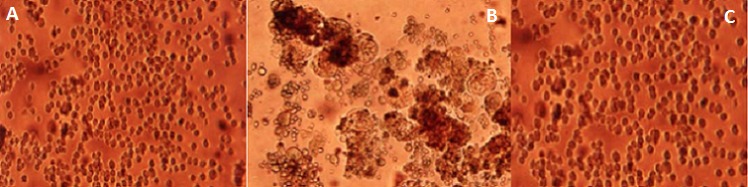
Inhibition of cytopathic effect [CPE] by the drugs. C8166 cells were plated in 96-well plates at a concentration of 1x10^5^/ml in RPMI1640 culture medium and infected with HIV-1025RTA at a MOI of 0.01 and various concentrations of ARVs ranging from 0 to 5µg/ml added. The micro plates were incubated for 3 to 5 days and cells observed daily for CPE under inverted microscope. A and C show inhibition of CPE by the ARVs at a concentration of 5µg/ml and mock infected control respectively. At 0.04µg/ml drugs concentration B a typical CPE of giant multinucleated cells is seen. Magnification x 400

The 50% inhibitory concentrations (IC_50_) of ARVs against all the HIV-1 strains were derived from the respective susceptibility curves (Figure 1). The ARV_S_ exhibited inhibitory activity against the two HIV-1 clinical isolates and the two laboratory strains with the IC50 ranging from 0.02 to 0.2 µg/ml within the normal ranges as previously reported [11]. The five ARVs exhibited a concentration dependent inhibition of HIV-1 in the two laboratory strains as well as in the two clinical isolates cytopathogenicity in C8166 cells. The 50% antiviral inhibitory concentration (IC_50_) of the ARVs is shown on Figure 1. The ARVs also Inhibited cytopathic effect (CPE) on C8166 cells (Figure 2), neither were they cytotoxic at concentrations used.

## Discussion

The antiviral activity of drugs can vary greatly because of factors such as genetic variation in isolates, host cell type, and multiplicity of infection assay used for measurement of virus replication. Because of genetic variation, determination of antiviral activity against a broad spectrum of viruses of well-characterized HIV laboratory strains and clinical isolates sufficient to assess the breadth of antiviral activity is recommended. In a previous study clade D viruses were reported to function with diminished drug sensitivity owing to rapid growth kinetics, whereas subtypes A, B, C and E demonstrated comparable sensitivity [12]. In the current study no decrease in sensitivity to AZT, Efavirenze, Abacavir, Lopinavir/Ritonavir and Nevirapine/Lamivudine/Stavudine was detected in the two HIV-1 clinical isolates and the Laboratory strains HIV-1_IIIB_ and HIV-1_JRFL_. The laboratory strain HIV-1IIIB was found to be less susceptible to antiretroviral drugs and this could be linked to the rapid growth of the strain. The decreased sensitivity noted in HIV-1IIIB probably owes to its high replication rate. The findings of this study concur with several previous reports that suggested similar *in vitro* susceptibilities to antiretroviral drugs among different group M subtypes [13, 14].

## Conclusion

In this study we have shown that non-B subtype isolates of HIV-1 are similar in their drug susceptibility to subtype B isolates. These findings suggest that the five anti-HIV compounds used in this study retain their anti-HIV activity against HIV-1 viral strains other than subtype B and continue being useful for the treatment of persons infected with non-B HIV-1 subtypes. Similar clinical benefits of antiviral therapy could therefore be anticipated in persons infected with subtypes of HIV-1 other than subtype B. The major limitations in this study are the few viruses studied and the fact that only five ARVs were assayed despite the wide spectrum of drugs in the three main classes.

### What is known about this topic

- Non-B subtype isolates of HIV-1 are similar in their drug susceptibility to subtype B isolates as has been previously reported.
- There are similar clinical benefits of antiviral therapy use in persons infected with subtypes of HIV-1 other than subtype B.

### What this study adds

- This is the first report of use of a phenotypic assay using a cultured virus to asses drug susceptibility of non B Kenyan HIV strains.
- This is first report of use of phenotypic assay to evaluate generic ARVs widely used in Kenya as well as in many other low and middle income countries.
- The results suggest a biochemical similarity of the reverse transcriptase (RT) and protease enzymes from the non B Kenyan subtypes despite the divergence at the genetic level.
